# Supplementary material for: Alanine-Dependent TCA Cycle Promotion Restores the Zhongshengmycin-Susceptibility in Xanthomonas oryzae
Source: Int J Mol Sci. 2023 Feb 3;24(3):3004. doi: 10.3390/ijms24033004 (PMC9918224; doi:10.3390/ijms24033004)
Supplement: Supplementary file 1 [file ijms-24-03004-s001.zip › ijms-2160149-supplementary.pdf]

Supplementary Materials

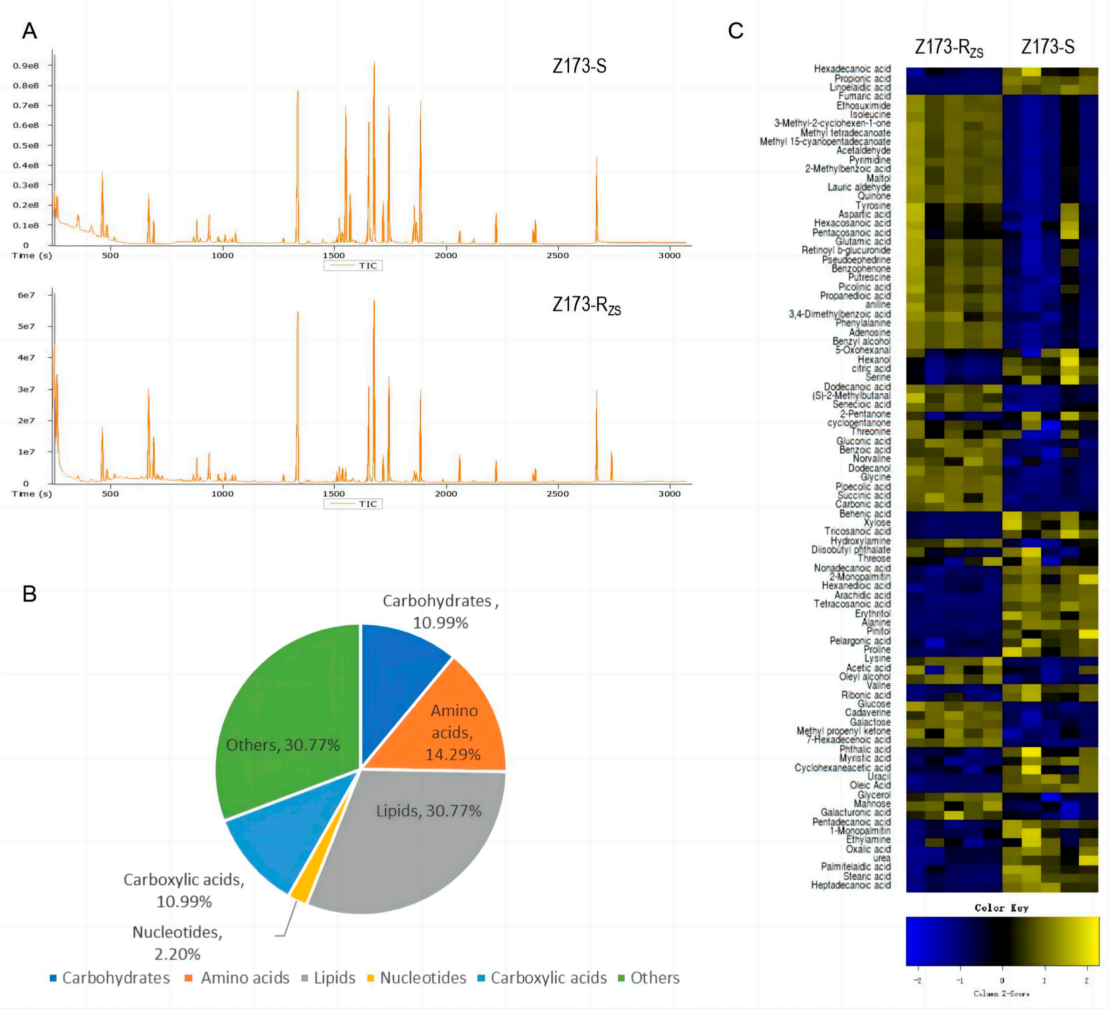

Fig. S1 Metabolomics analysis of Z173-S and Z173-R$_{ZS}$. (A) representative total ion current chromatogram of Z173-S and Z173-R$_{ZS}$; (B) Category of metabolites detected. (C) Heat map of unsupervised hierarchical clustering of metabolites detected. Yellow and blue indicate increase and decrease of metabolites relative to the median metabolite level, respectively(see color scale).

Table S1. The primers used in this study for qRT-PCR.

Primers	Sequences (5'-3')	Annotation
qRT-16s-F<br>qRT-16s-R	AACACTGACACTGAGGCACGAAAG<br>CCCAGGCGGCGAACTTAACG	16S-*Xanthomonas oryzae* strain LMG 5047 16S ribosomal RNA
OGDH-E1-F<br>OGDH-E1-R	GCACGACCAGAAGACCGACAAC<br>CCATCACCGCTTCTTCGCTGAG	2-oxoglutarate dehydrogenase, E1 component
OGDH-E2-F<br>OGDH-E2-R	GCCAGTCGTTCGCCGATGTC<br>AAGGTGCCGCCATTGGTGATG	2-oxoglutarate dehydrogenase, E2 component
SDH-hmap-F<br>SDH-hmap-R	GCAGGTGGTGCTGGAAGATTACG<br>ACGGCAAACACGCTGACGATG	Succinate dehydrogenase, hydrophobic membrane anchor protein
SDH-fs-F<br>SDH-fs-R	GCTGCTGGATCTGGTGGTGTTC<br>AAGGTCTTGTGCGGCTGATTGG	Succinate dehydrogenase, flavoprotein subunit
SDH-b556-F<br>SDH-b556-R	CGTTCGTCGGCATGGGTTGG<br>TACGGCACCTCCAGACAGCAG	Succinate dehydrogenase, cytochrome b556 subunit
SDH-iron-F<br>SDH-iron-R	TGAACATCGACGGCACCAATACG<br>GCAGCGGATAGATCGGCACTTC	Succinate dehydrogenase iron-sulfur protein
PDH-cda-F<br>PDH-cda-R	AGAGCCTGGTCACCCTGGAATC<br>ACCACACTGCCCTGCGAGAG	Pyruvate dehydrogenase complex dihydrolipoamide acetyltransferase
PDH-E1-F<br>PDH-E1-R	CGTGCCTTTGCTGACCAGATCC<br>TCCGAGCGACCGAACCCATC	PDH E1component
PDH-c-F<br>PDH-c-R	CGGCAAGGAATACGTGGAGTGG<br>AAGGTGTCGCAGTTGAGCATCG	PDH cytochrome
